# Does Extending the Waiting Time of Low-Rectal Cancer Surgery after Neoadjuvant Chemoradiation Increase the Perioperative Complications?

**DOI:** 10.1155/2016/7870815

**Published:** 2016-09-22

**Authors:** Kittinut Timudom, Natthawut Phothong, Thawatchai Akaraviputh, Vitoon Chinswangwatanakul, Ananya Pongpaibul, Janjira Petsuksiri, Suthinee Ithimakin, Atthaphorn Trakarnsanga

**Affiliations:** ^1^Department of Surgery, Faculty of Medicine, Siriraj Hospital, Mahidol University, Bangkok, Thailand; ^2^Department of Surgery, Panyananthaphikkhu Chonprathan Medical Center, Srinakharinwirot University, Nonthaburi, Thailand; ^3^Department of Pathology, Faculty of Medicine, Siriraj Hospital, Mahidol University, Bangkok, Thailand; ^4^Department of Radiology, Faculty of Medicine, Siriraj Hospital, Mahidol University, Bangkok, Thailand; ^5^Division of Medical Oncology, Faculty of Medicine, Siriraj Hospital, Mahidol University, Bangkok, Thailand

## Abstract

*Background.* Traditionally, rectal cancer surgery is recommended 6 to 8 weeks after completing neoadjuvant chemoradiation. Extending the waiting time may increase the tumor response rate. However, the perioperative complication rate may increase. The purpose of this study was to determine the association between extending the waiting time of surgery after neoadjuvant chemoradiation and perioperative outcomes.* Methods*. Sixty patients with locally advanced rectal cancer who underwent neoadjuvant chemoradiation followed by radical resection at Siriraj hospital between June 2012 and January 2015 were retrospectively analyzed. Demographic data and perioperative outcomes were compared between the two groups.* Results*. The two groups were comparable in term of demographic parameters. The mean time interval from neoadjuvant chemoradiation to surgery was 6.4 weeks in Group A and 11.7 weeks in Group B. The perioperative outcomes were not significantly different between Groups A and B. Pathologic examination showed a significantly higher rate of circumferential margin positivity in Group A than in Group B (30% versus 9.3%, resp.; *P* = 0.04).* Conclusions*. Extending the waiting to >8 weeks from neoadjuvant chemoradiation to surgery did not increase perioperative complications, whereas the rate of circumferential margin positivity decreased.

## 1. Background

In Thailand, colorectal cancer is the third most common cancer after liver and lung cancer in men and the fifth most common cancer after cervix, breast, liver, and lung cancer in women. Approximately one-third of Thai patients with colorectal cancer have metastatic disease [1]. Neoadjuvant chemoradiation (nCRT) followed by total mesorectal excision (TME) is the preferred treatment for the locally advanced rectal cancer (clinical stage T3 or T4 or node-positive disease). This treatment regimen has many benefits, including improved local control, reduced toxicity, and an increased incidence of sphincter-salvage operations [2, 3]. In 2004, Sauer et al. [2] published a randomized study of 799 patients with locally advanced rectal cancer. These patients were divided into a preoperative long-course chemoradiotherapy group and a postoperative chemoradiotherapy group. The authors found that preoperative chemoradiotherapy followed by radical surgery with TME 6 weeks after the completion of chemoradiotherapy was associated with an improved tumor response rate, reduced chemoradiotherapy-related toxicity, decreased 5-year local recurrence rate, and increased sphincter preservation rate. Nonetheless, the 5-year disease-free survival and overall survival rates were not significantly different from those in the postoperative treatment group.

The optimal time between nCRT and surgery is still widely disputed. In 1999, Francois et al. [4] demonstrated that a long-interval group (6 to 8 weeks) from preoperative chemoradiation to surgery was associated with significantly increased pathologic downstaging (10.3% versus 26%, resp.; *P* = 0.005) and a nonsignificant improvement in the rate of sphincter-preserving surgery (68% versus 76%, resp.; *P* = 0.27) compared to an interval of 2 to 3 weeks. Later, several studies proposed that increased time intervals of >8 weeks might improve tumor downstaging base on the time-dependent response of the radiation effect [5–10]. However, many surgeons are concerned about delaying surgery for >8 weeks because the effects of a longer interval on both perioperative complications and oncologic outcomes remain unclear [5, 9]. The objective of this study was to evaluate the association between extending the waiting time of surgery after nCRT and perioperative outcomes.

## 2. Materials and Methods

Sixty patients with locally advanced low-rectal cancer (defined as a tumor ≤ 7 cm from the anal verge) who underwent nCRT followed by radical surgery (17 patients underwent surgery ≤8 weeks and 43 patients underwent surgery >8 weeks after nCRT) between June 2012 and January 2015 were consecutively selected by surgeon preferences. Data were obtained from the patients' medical records. Clinical staging was performed by computed tomography, colonoscopy, and endorectal ultrasound. Patients with evidence of distant metastasis on preoperative imaging were excluded. Demographic data collected included age, body mass index (BMI), sex, Charlson comorbidity index, clinical T and N stages, and distance of the tumor from the anal verge. All patients underwent nCRT comprising 45.0 to 50.4 Gy in 28 fractions with a continuous infusion of 5-fluorouracil (1000 mg/m^2^ per day) for 5 days during the first and fifth week of radiation therapy.

The patient underwent surgery after completing nCRT at various intervals of 4 to 13 weeks depending on the clinical response, scheduling, and surgeon's preference. Surgical procedures were performed by experienced surgeons in the colorectal unit and minimally invasive surgery unit of the department of surgery, Siriraj Hospital. The oncologic principals of total mesorectal excision and high vessel ligation were utilized. Gastrointestinal pathologists examined the surgical specimens. A pathologic complete response (pCR) was defined as a complete absence of tumor cells in the resected specimen (ypT0) and the resected nodes (ypN0). Circumferential margins were reported to be involved if microscopic tumor cells were present ≤1 mm from the margins. Operative and postoperative data including the type of operation, rate of diverting stoma formation, operative time, estimated blood loss, blood transfusion, time to bowel movement, time to full diet intake, postoperative length of hospital stay, and postoperative complications (Clavien-Dindo classification) were recorded.

### 2.1. Statistical Analysis

Statistical analysis of all collected data was performed with SPSS statistical software version 19.0 (IBM Corporation, Armonk, NY, USA). Categorical variables are expressed using frequency. Numerical variables are expressed as means with 95% confidence interval. Statistical analysis was performed with Fisher's exact test and the chi-square test, where appropriate. A two-sided *P* value of <0.05 was considered statistically significant.

## 3. Results

Sixty patients with locally advanced low-rectal cancer underwent nCRT followed by radical resection (both sphincter-preserving low anterior resection and abdominoperineal resection) with TME. The patients were divided into two groups. In Group A, 17 patients underwent surgery ≤8 weeks after nCRT; in Group B, 43 patients underwent surgery >8 weeks after nCRT. The mean interval after completion of nCRT was 6.4 weeks in Group A and 11.7 weeks in Group B.

The patients' demographic variables including age, BMI, sex, Charlson comorbidity index, clinical T and N stages, and distance of the tumor from the anal verge were not significantly different between the two groups (Table 1).

Operative data are shown in Table 2. There were no significant differences in the rate of sphincter-preserving surgery or diverting stoma formation between the two groups (41% versus 55%, *P* = 0.30; and 100% versus 87%, *P* = 0.78, resp.).

The perioperative results are shown in Table 3. The operative time, estimated blood loss, units of blood transfusion, return of bowel function, and postoperative length of stay were not significantly different between the two groups.

The postoperative complication rate according to the Clavien-Dindo classification was 11.7% in Group A (bowel ileus, *n* = 1; stomal necrosis, *n* = 1) and 18.6% in Group B (*P* = 0.19). The most frequent complication in Group B was bowel ileus (62.5%). The others were anastomosis leakage (*n* = 1), wound infection (*n* = 1), and stomal necrosis (*n* = 1) (Table 4).

The pathological outcomes are shown in Table 5. There was a significant difference in the number of patients with circumferential margin positivity between Groups A and B (30% versus 9.3%, resp.; *P* = 0.04). Furthermore, there was lower proportion of patients in Group A than B who obtained a pCR (11.7 versus 18.6%, resp.; *P* = 0.52). Tumor grading and perineural and lymphovascular invasion were similar in both groups.

## 4. Discussion

In this recent study, the postoperative complication rate according to the Clavien-Dindo classification in the long-interval group (>8 weeks) was not significantly different from that in the short-interval group (≤8 weeks) (18.6% versus 11.7%, resp.; *P* = 0.19). The most common complication in the long-interval group was bowel ileus, and all cases were successfully managed with conservative treatment. This result answered question of many surgeons who are concerned that a longer waiting period of ≥8 weeks may lead to greater intraoperative difficulty because of radiation-induced fibrosis [9, 11]. Moore et al. [12] found that a mean interval time of >44 days from preoperative nCRT to surgery was associated with a trend toward a higher rate of anastomotic complications compared to a shorter interval (7% versus 0%, resp.; *P* = 0.05). Wong et al. [13] reviewed randomized trials that compared the results of preoperative radiotherapy with those of surgery alone or other neoadjuvant or adjuvant strategies. They found that preoperative radiotherapy improved the local recurrence rate but increased the risk of wound infection following surgery and had long-term effects on rectal and sexual function. Another study showed that a longer waiting period of ≥8 weeks was not associated with increased rates of postoperative complications, morbidity and mortality, or diverting stoma formation [14].

However, a limitation of this study is that it was a retrospective, single-center study, and it had low overall number of the patients especially in short-interval group. This evidently limited the power of the study to detect differences. Moreover, the individual surgeon decided the time interval between chemoradiation and surgery. This may have introduced potential bias in association with the retrospective design. Another potential limitation is that longer-term outcomes were not evaluated. A higher-quality study design and larger number of patients will more strongly emphasize the power of outcomes.

In terms of pCR, we found that the pCR rate had a tendency to be higher in the long-interval group (>8 weeks) than in the short-interval group (≤8 weeks) from this study (18.6% versus 11.7%, resp.; *P* = 0.52). This is in concordance with many reports, which have encouraged extending the waiting time to ≥8 weeks before surgery. Kalady et al. [15] revealed that a time interval of ≥8 weeks from nCRT to surgery was the only independent predictor of pCR. Sloothaak et al. [6] performed a retrospective study of a large number of patients and showed that delaying surgery 10 to 11 weeks after the completion of nCRT is associated with increased nodal downstaging and a higher pCR rate. However, delaying surgery to >11 weeks was not associated with superior oncologic outcomes. Likewise, a study by Probst et al. [16] showed that the peak cumulative pCR rate was associated with an interval time ranging from 10 to 11 weeks, and no association with 30-day mortality was observed among 17,255 patients with stage II and III rectal cancer. Additionally, the rate of circumferential margin positivity was significantly lower in the long- than short-interval group (9.3% versus 30.0%, resp.; *P* = 0.04). The above-described prior studies indicate that extending the waiting time to >8 weeks has no impact on the sphincter preservation rate. Our data support this result in that neither the rate of sphincter-preserving surgery nor the rate of diverting stoma formation was significantly different between the long-interval group (>8 weeks) and the short-interval group (≤8 weeks) (*P* = 0.30 and *P* = 0.78, resp.).

## 5. Conclusions

Our findings suggest that extending the waiting time of rectal cancer surgery after nCRT to ≥8 weeks does not increase perioperative complications, including the rate of diverting stoma formation. Although we were unable to demonstrate a statistically significant improvement in the rate of pCR by extending the waiting time to ≥8 weeks, the rate of circumferential margin positivity was significantly decreased using this time interval. Thus, extending the waiting time after nCRT may be a safe technique that does not change perioperative outcomes.

## Figures and Tables

**Table 1:** Demographic data.

Characteristic	Group A (≤8 weeks) *n* = 17	Group B (>8 weeks) *n* = 43	*P* value
Age (years)	53.2 (45.6–60.8)	61.9 (67.5–66.3)	0.37
Body mass index (kg/m^2^)	22.0 (19.5–24.6)	23.8 (22.5–25.1 )	0.17
Sex			0.38
Male	14 (82)	23 (53)	
Female	3 (28)	20 (47)	
Mean Charlson comorbidity index	1.2	1.4	0.61
Clinical T stage			0.89
T3	14 (82)	36 (83)	
T4	3 (18)	7 (17)	
Clinical N positive			0.31
Negative	2 (12)	10 (23)	
Positive	15 (88)	33 (76)	
Distance from anal verge (cm)	4.5 (3.4–5.7)	5.6 (4.9–6.3)	0.17

Except for the mean Charlson comorbidity index, values are presented as mean (95% confidence interval) or number (%).

**Table 2:** Operative data.

Variable	Group A (≤8 weeks) *n* = 17	Group B (>8 weeks) *n* = 43	*P* value
Operation			0.30
Sphincter-preserving LAR	7 (41)	24 (55)	
APR	10 (58)	19 (45)	
Diverting stoma			0.78
Colostomy	2 (29)	8 (33)	
Ileostomy	5 (71)	13 (54)	
No diversion	0 (0)	3 (13)	

Values are presented as number (%).

**Table 3:** Perioperative results.

Variable	Group A (≤8 weeks) *n* = 17	Group B (>8 weeks) *n* = 43	*P* value
Operative time (min)	277 (234–320)	255 (223–286)	0.43
Estimated blood loss (mL)	374 (196–551)	360 (239–481)	0.90
Blood transfusion (units)	0.4 (0.0–0.9)	0.3 (0.4–0.5)	0.50
Time to bowel movement (days)	3.0 (2.3–3.6)	3.3 (2.7–4.0)	0.51
Time to full diet intake (days)	4.0 (3.0–5.0)	3.7 (3.1–4.2)	0.58
Postoperative LOS (days)	8.0 (6.0–10.1)	8.6 (6.0–11.1)	0.79

Values are presented as mean (95% confidence interval).

**Table 4:** Postoperative complications.

Clavien-Dindo classification	Group A (≤8 weeks) *n* = 17	Group B (>8 weeks) *n* = 43	*P* value
Grade 1	0	1	
Grade 2	1	6	
Grade 3A	0	0	
Grade 3B	1	1	
Grade 4	0	0	
Grade 5	0	0	

Total	2 (11.7%)	8 (18.6%)	0.19

Values are presented as number (%).

**Table 5:** Pathological results.

Tumor characteristics	Group A (≤8 weeks)	Group B (>8 weeks)	*P* value
	*n* = 17	*n* = 43	
Tumor grading			
Well-differentiated	1 (5.9)	1 (2.3)	
Moderately differentiated	14 (82.3)	35 (81.3)	
Poorly differentiated	2 (11.8)	2 (4.7)	0.39
Circumferential margin positivity	5 (30)	4 (9.3)	0.04
Invasion			
Perineural invasion	7 (41.1)	16 (37.2)	0.77
Lymphovascular invasion	2 (11.7)	8 (18.6)	0.52
Pathologic complete response	2 (11.7)	8 (18.6)	0.52

Values are presented as number (%).

## Abbreviations

nCRT: Neoadjuvant chemoradiation
TME: Total mesorectal excision
BMI: Body mass index
pCR: Pathologic complete response
DFS: Disease-free survival
OS: Overall survival
LAR: Low anterior resection
APR: Abdominoperineal resection
LOS: Length of hospital stay.
